# Carbohydrate Taste Is Associated with Food Intake and Body Mass in Healthy Australian Adults

**DOI:** 10.3390/nu13113844

**Published:** 2021-10-28

**Authors:** Andrew Costanzo, Natwalinkhol Settapramote, Niramon Utama-ang, Uracha Wanich, Simone Lewin, Russell Keast

**Affiliations:** 1 CASS Food Research Centre, School of Exercise and Nutrition Sciences, Deakin University, Melbourne, VIC 3125, Australia; andrew.costanzo@deakin.edu.au (A.C.); uracha.w@rbru.ac.th (U.W.); simone.lewin@deakin.edu.au (S.L.); 2 Department of Agro-Industry, Faculty of Agriculture and Technology, Rajamangala University of Technology Isan Surin Campus, Surin 32000, Thailand; natwalinkhol.se@rmuti.ac.th; 3 Division of Product Development Technology, Faculty of Agro-Industry, Chiang Mai University, Chiang Mai 50200, Thailand; niramon.u@cmu.ac.th; 4 Cluster of High Value Product from Thai Rice for Health, Faculty of Agro-Industry, Chiang Mai University, Chiang Mai 50200, Thailand

**Keywords:** carbohydrate, taste, alimentary, body mass, test-retest

## Abstract

Background: The taste of carbohydrates may drive their intake. Sensitivity to carbohydrate taste varies among individuals, thus, it is important to understand how differences in sensitivity influence eating behaviour and body mass. Objective: The aims of this study were to assess associations among carbohydrate taste sensitivity, habitual and acute food intake, and body mass; as well as assess the reliability of the carbohydrate detection threshold (DT) test within and across days. Methods: Carbohydrate DT was assessed six times across three sessions in 36 healthy adult participants (22 female) using a three-alternate forced choice methodology. Moreover, 24 h diet records were completed on the days prior to testing sessions, and food intake at a buffet lunch was collected following each session. Anthropometry was also measured. Linear mixed regression models were fitted. Results: The DT test required at least three measures within a given day for good reliability (ICC = 0.76), but a single measure had good reliability when compared at the same time across days (ICC = 0.54–0.86). Carbohydrate DT was associated with BMI (kg/m^2^: β = −0.38, *p* = 0.014), habitual carbohydrate intake (g: β = −41.8, *p* = 0.003) and energy intake (kJ: β = −1068, *p* = 0.019) from the 24-h diet records, as well as acute intake of a buffet lunch (food weight (g): β = −76.1, *p* = 0.008). Conclusions: This suggests that individuals who are more sensitive to carbohydrate are more likely to consume greater quantities of carbohydrates and energy, resulting in a greater body mass.

## 1. Introduction

Obesity is a threat to human health because it increases the risk of major causes of mortality, including heart disease, diabetes, hypertension, stroke, dyslipidaemia, and certain cancers [1]. In simple terms, obesity is a result of energy intake exceeding energy expenditure over a prolonged period. Part of the reason why individuals may consume excess energy is attenuated fullness after consuming a meal which creates the need to continue consumption. Satiety is the feeling of fullness that persists after eating, potentially suppressing further energy intake until hunger returns [2]. If the satiety cascade is extended after a meal, then subsequent food intake may be decreased [3,4]. In terms of satiation, or stopping eating during a meal, it is likely that perceptual factors are of primary importance due to their dominant role prior to ingestion [5,6,7].

The basic tastes (sweet, sour, salt, and bitter) with their perceptual salience and associated hedonic response have a primary role as a gatekeeper for ingestion. More recent evidence indicates that newly identified tastes such as fat, umami, and kokumi have a key role post-ingestion with links to satiety [8]. These putative tastes appear to be linked with macronutrient sensing and have been termed “alimentary tastes” [9]. Carbohydrate taste is one of these putative tastes and there is emerging research illustrating an association between carbohydrate taste and consumption. In a recent study, Low et al. reported participants who were classified as hypersensitive to carbohydrate taste (which was determined as the oral detection threshold (DT) to maltodextrin), consumed significantly more maltodextrin-based milkshake in comparison to hyposensitive participants [10]. This increased consumption was independent of liking. Earlier studies by Low et al. reported that participants who were hypersensitive to carbohydrate taste consumed a greater quantity of foods containing complex carbohydrates and had a larger waist circumference [11,12].

The current standard for assessing carbohydrate taste DT is to use an ascending forced choice triangle methodology. Low et al. [13,14] assessed the test-retest reliability of this methodology and reported that it was reliable across seven repeated measures for maltodextrin (ICC = 0.95) and oligofructose (ICC = 0.95) [15]. However, this study did not account for how many tests were conducted within a single day or the time of day. In other words, some or all of the seven repeated measures may have been conducted on the same day or across different days and times, and this structure was not consistent between participants. Diurnal rhythms have been shown to influence fat, sweet and salty taste sensitivities [16,17,18]; thus, it is crucial that test-retest assessments are conducted to control for time of day.

The aims of the present study were to assess associations between carbohydrate taste sensitivity, habitual and acute food intake, perceptions of satiety, and body mass; as well as assess the reliability of the carbohydrate DT test within and across days.

## 2. Methods

### 2.1. Participants

Forty-two participants were recruited by flyers on notice boards around Deakin University, Burwood campus, Australia. Exclusion criteria included individuals who were: smokers, were pregnant or lactating, were taking any prescription medication that may interfere with their ability to taste, had any food allergies or intolerances, or were unable to safely fast overnight due to health problems. Participants provided informed consent before the experiment was conducted, and the protocol was approved by the Deakin University Faculty of Health Human Ethics Advisory Group (HEAG-H 44_2018).

### 2.2. Study Design

This study was part of a larger study to assess the effect of three different carbohydrate-based drinks on satiety (currently unpublished) which has been retrospectively registered with the Australian New Zealand Clinical Trials Registry (Registration ID: ACTRN12619000747123). Participants visited the CASS Food Research Centre laboratory at Deakin University in Burwood on five separate occasions, with at least 24 h between each session. Data were collected using Compusense Cloud software as part of the Compusense Academic Consortium (v21, Compusense Inc., Guelph, ON, Canada) in isolated sensory booths.

In laboratory session 1, anthropometry was measured, and participants rated the suprathreshold (ST) taste intensity for six tastants (four basic tastes, umami, and carbohydrate taste). Session 5 followed the same process as session 1 with the addition of completing the Three Factor Eating Questionnaire (TFEQ) [19].

**Figure 1:**
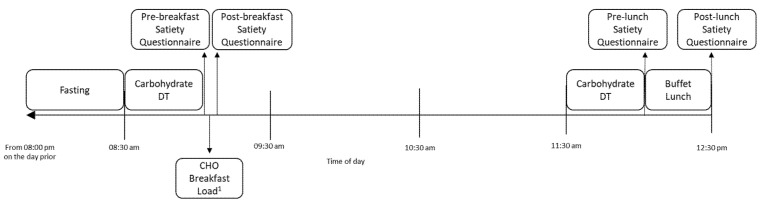
Study timeline for sessions 2–4.

For sessions 2–4 (Figure 1), participants were asked to fast from 8:00 p.m. the night prior to the laboratory session, which was confirmed upon arrival to the laboratory. Participants attended the laboratory at 8:30 a.m. on the testing day, were assessed for carbohydrate taste DT and rated their satiety on a questionnaire (pre-breakfast). One of three carbohydrate beverages was provided as breakfast at random. Initially, participants were provided with 200 mL preload of the beverage, which they were required to consume in its entirety within 5 min. Then, participants were asked to consume the same beverage *ad libitum* until they were comfortably full, after which they completed another satiety questionnaire (post-breakfast). Participants were then able to leave the lab and were asked to refrain from eating or drinking anything other than water until their return to the lab at 11.30 a.m. or after 3 h, when carbohydrate DT was assessed again. Participants completed a satiety questionnaire (pre-lunch) and were then provided an *ad libitum* buffet lunch. The lunch was consumed within a maximum period of 1-h and a final satiety questionnaire was completed afterwards (post-lunch).

### 2.3. Detection Threshold for Carbohydrate Taste

Twelve concentrations of oligofructose solutions were prepared using previous methods [12]. Oligofructose has been shown to be an effective carbohydrate tastant with high correlation to other carbohydrate tastants such as maltodextrin [12]. DT was determined using a validated ascending forced choice triangle methodology [14,20] in which the participants were provided with three 25 mL samples in cups labelled with randomised three-digit blinding codes, two of which were controls (filtered deionised water) and one containing oligofructose solution from 0.4 to 200 g/L [12]. The order in which samples within set of three was randomised throughout the procedure. Participants were asked to rinse their mouths with water before beginning the task, with a 60 s interval between sample sets. Participants wore nose clips, and all tests were conducted under red light. Participants were tasked with identifying the “odd” sample in the set. Correct identification of the oligofructose solution resulted in the participants repeating the same sample set. Incorrect identification of the oligofructose solution sample resulted in a new sample set with a higher concentration of oligofructose solution. This continued until participants were able to correctly identify three sample sets in a row, and this concentration was determined to be their DT.

DT was converted from concentration of oligofructose (g/L) to an ordinal variable based on concentration step used in the test (i.e., 0.4 g/L = concentration step 1, 200 g/L = concentration step 12). This was to normalise the data and reduce the orders of magnitude in differences seen between the higher concentration steps, as conducted in previous studies [21]. The mean of the two DT measures from each session (pre-breakfast and pre-lunch) was used for all statistical analyses unless stated otherwise.

### 2.4. Suprathreshold Ratings

Solutions were prepared at ST concentrations for sweet (sucrose, Woolworths, Bella Vista, NSW, Australia), salty (sodium chloride, Woolworths, Bella Vista, NSW, Australia), sour (citric acid, Ward McKenzie, Altona, VIC, Australia), bitter (caffeine, Sigma-Aldrich, St. Louis, MO, USA), umami (monosodium glutamate (MSG), Ajinomoto Cooperation, Tokyo, Japan), and carbohydrate (oligofructose, Fibrulose F97, Cosucra Groupe Warcoing, Belgium via Brenntag Australia Pty. Ltd., Melbourne, VIC, Australia) tastes according to previous methods [15,22]. These taste measures were included as control measures to assess normal taste function, as the evidence for carbohydrate taste function is still in its infancy. Participants rated the intensity of three concentrations (weak, medium, and strong) for the four basic tastes and umami, as well as four concentrations (weak, medium, medium–strong, and strong) for carbohydrate taste (Table 1). A control (blank) sample was rated alongside all tastants. Participants wore nose clips, and the test was conducted under red light to prevent confounding non-taste sensory cues. They tasted each sample and rated the intensities on a labelled magnitude scale (LMS) with anchors 0 (no taste) to 100 (strongest imaginable taste). All solutions were prepared on the day of testing, served at room temperature.

### 2.5. Preparation for Carbohydrate Breakfast Beverage

The carbohydrate breakfast beverages are not part of the aims described in this manuscript. They were provided to participants as part of a larger study outside of the scope of this manuscript. However, it is important to consider these beverages as potential confounders of the outcomes described in this manuscript, specifically, perceived satiety and *ad libitum* buffet lunch intake. Therefore, the breakfast beverages are described here for full disclosure and were included as potential covariates in the relevant statistical analyses.

Jasmine rice powder (made from whole grain of white rice from northern Thailand), riceberry rice powder (made from whole grain of riceberry rice from northern Thailand), and oligofructose powder were the carbohydrate bases for each beverage. Each beverage consisted of hot water, one carbohydrate powder (6.31% *w*/*v*), sucralose (0.0025% *w*/*v*) (Splenda powder, Victoria, Australia), NaCl (0.2% *w*/*v*) (Woolworths, Bella Vista, NSW, Australia), and vanilla flavour (0.000075% *v*/*v*) (Specialty Flavours & Fragrances, Victoria, Australia). The ingredients were mixed at 10,000 rpm for 2 min using a homogeniser (Silverson, model L4R, England). The energy content of the beverages made from riceberry and jasmine rice was similar (95.5 and 90.0 kJ per 100 mL, respectively) and the oligofructose beverage contained negligible energy content due to it being predominantly fibre.

### 2.6. Perceived Satiety Measurements

The satiety questionnaire used 100 mm visual analogue scales (VAS) where 0 mm represented “not at all” and 100 mm represented “extremely”, in response to the questions “How full are you?” and “How hungry are you?” [23]. The differences between pre-breakfast and post-breakfast satiety scores and pre-lunch and post-lunch satiety scores were calculated.

### 2.7. Ad Libitum Buffet Lunch Intake

The buffet lunch was served in a social setting with participants eating together for ecological validity, and they were asked to eat until comfortably full. Lunch was provided in excess (7927 kJ per person), and foods included hot potato chips (Simplot, Mentone, Australia) (150 g), potato and leek soup (Continental, New South Wales, Australia) (250 g), pasta bake (pasta: San Remo, Windsor Gardens, South Australia; sauce: Mars, Auckland, New Zealand) (425 g), nachos (corn chip: Coles, Victoria, Australia; cheese: Coles, Victoria, Australia and mild salsa: Doritos, UK) (150 g), garden salad with French salad dressing (Praise, Dunedin, New Zealand) (120 g), tinned peaches (SPC Ardmona, Victoria, Australia) (150 g), sliced apple (100 g), chocolate bar (Mars, Auckland, New Zealand) (34 g), tomato sauce (Masterfoods, Auckland, New Zealand) (15 g), thickened cream (Coles, Victoria, Australia) (20 g), and tap water (300 g) [24]. Additional servings of foods were provided to participants upon request. The quantity of individual foods consumed was calculated via weight (g food supplied − g food returned = g food consumed). The mean energy intake (kJ) and quantity (g) of dietary fat, protein and carbohydrate (including total carbohydrate, sugar and starch) consumed were calculated using FoodWorks software (v8, Xyris software, Queensland, Australia).

### 2.8. Habitual Dietary Intake

Participants completed three 24-h diet records, one for each day preceding sessions 2, 3 and 4. If a participant had two sessions on consecutive days, the record for the latter day was conducted on a separate weekday, chosen by the participant, within the same week of testing, so as to not have been influenced by fasting or the *ad libitum* buffet lunch from the study. Sessions never occurred on Mondays or weekends, thus, all records were completed on weekdays. Participants were taught to quantify foods in standard serving sizes (cups, teaspoons, tablespoons, etc.) with the use of a food model booklet and asked to provide the weight or volume of their food and drinks wherever possible. Details such as brand, cooking method, and food additives (e.g., sugar added to coffee) were included in the diet records. 

Diet records were analysed for energy intake (kJ) and quantity (g) of dietary fat, protein, and carbohydrate (including total carbohydrate, sugar, and starch) consumed using FoodWorks software. The records were then assessed for underreporting using Goldberg cut-off values [25]. If the ratio of a participant’s reported energy intake to basal metabolic rate (based on age, sex and weight) was lower than 0.9, then the energy intake in that food record was not considered to be reflective of their usual intake and was excluded from analysis. The nutrition data from the diet records for each participant were aggregated to estimate habitual food intake, as three 24-h diet records are found to be sufficient for estimating habitual energy and macronutrient intakes [26].

### 2.9. Anthropometry

Participants were asked to remove shoes, heavy clothing and pocket contents before weight and height were measured using dedicated digital scales (Tanita Body Scan Composition Monitor Scales, Cloverdale, WA, Australia) and a portable stadiometer (Seca, MedShop Australia, Fairfield, VIC, Australia). BMI was calculated as weight (kg)/height (m)^2^. Waist and hip circumferences were measured using an ergonomic circumference measurement tape as per World Health Organisation guidelines [27].

### 2.10. Three-Factor Eating Questionnaire

Participants completed the TFEQ [19] by answering 51 questions relating to their eating habits and feelings towards eating. The questionnaire consists of three distinct constructs, although only one construct was used in this study—cognitive restraint. Participants were identified as restricted eaters (RE) if they received a score of greater than 12 on the cognitive restraint construct [24].

## 3. Statistical Analysis

Statistical analyses were conducted using IBM SPSS statistical software version 26.0 (SPSS, Chicago, IL, USA). Data are presented as mean (standard deviation) unless stated otherwise. Significance was accepted at *p* < 0.05.

Intraclass correlation (ICC) estimates and their 95% confidence intervals (CI) were calculated on the basis of an absolute-agreement, two-way mixed-effects model [28]. ICC analysis was used to assess reliability within the duplicate measures of each tastant for the six ST intensity ratings, and to test reliability within and between testing days for carbohydrate DT. The model parameters for the carbohydrate DT analysis were based on previous works [29]. The ICC values were interpreted as poor (<0.50), moderate (0.50–0.75), good (0.75–0.9), and excellent (>0.9) [28]. A paired *t*-test was used to assess any differences between pre-breakfast and pre-lunch carbohydrate DT measures.

Linear mixed models were used to assess the relationship between carbohydrate taste DT and anthropometry, demographics, habitual diet, breakfast beverage intake, buffet lunch intake and satiety ratings. All models were set with DT and session number (2–4) as fixed variables and individual subjects set as a random variable to control for intra-subject variation in the model. Covariates were also set as fixed variables, including their interactions with DT, if they had a significant mediating effect on the relationship between carbohydrate taste DT and the variable in question. Estimate sizes (β), 95% CIs and *p*-values from these models are reported.

## 4. Results

### 4.1. Participants

Of the 42 participants recruited into the study, six participants dropped out during testing, resulting in 36 participants completing the study. Twenty-two (61%) of the participants who completed the study were female. Demographic and anthropometric results are reported in Table 2. A post-hoc power calculation was performed using G *Power software (Release 3.1.9.7, Heinrich-Heine-Universität, Düsseldorf) to determine if enough participants were recruited to accurately measure carbohydrate DT according to Low et al. [12]. Using the following inputs: α = 0.05; oligofructose DT in Low et al.: 18.0 (23.3) g/L; oligofructose DT in the current study: 30.4 (36.3) g/L, a power of 89.1% was achieved.

### 4.2. Test-Retest Reliability of Carbohydrate Taste Detection Threshold

There was no significant difference observed between pre-lunch DT and pre-breakfast DT (mean diff = 0.37 steps, *p* = 0.225). Overall, the six measures of DT had good reliability (Table 3). For session 2 only (the first two measures of carbohydrate taste DT), the within day agreement between the pre-breakfast and pre-lunch DT was poor. When measures from sessions 3 and 4 were added to the model, agreement between the pre-breakfast and pre-lunch DT was moderate and good, respectively, suggesting that single measures of DT are unreliable. There was moderate to good agreement between DT measures for the same time conducted across days, indicating that repeating the test at the same time on separate days likely resulted in reliable results. Conversely, reliability between the two measures of carbohydrate ST intensity was poor while the other tastes ranged from poor to good (Table 1).

### 4.3. Anthropometric and Demographic Associations

Average carbohydrate taste DT was significantly associated with hip circumference (β = −0.87, *p* = 0.006), body weight (β = −1.65, *p* = 0.006) and BMI (β = −0.38, *p* = 0.014), where more sensitive individuals were more likely to have greater body mass. This association was not significant for waist circumference (β = −0.87, *p* = 0.092), although the trend was in the same direction as other measures of body mass.

### 4.4. Habitual Dietary Intake

Out of a total possible 108 diet records (three per participant), 25 were excluded due to being below the Goldberg cut-off value [25] of 0.9 and 21 were incomplete or not submitted, resulting in a total of 62 diet records being included in the analysis. Mean dietary intakes are displayed in Table 4.

In the base model, no significant associations between carbohydrate taste DT and habitual food consumption based on the self-reported diet records were observed. However, this was not the case when taking RE status into account in the model as a covariate. Seven participants (six females) were classified as REs according to the TFEQ. Firstly, there was no significant difference in energy intake between participants who were classified as REs and non-REs. When RE status was included in the model, there was a significant effect of RE status and the carbohydrate DT × RE interaction for all nutrient variables (*p* < 0.050). Therefore, RE status and the interaction with carbohydrate DT were included in the final model.

In the final model, significant associations were observed between carbohydrate DT and energy, total carbohydrate, and sugar intakes, along with a trend toward being associated with starch intake, and no associations with fat or protein intakes (Table 5).

### 4.5. Breakfast Beverage Intake

There was no association between carbohydrate DT and *ad libitum* intake of the carbohydrate breakfast beverage. Furthermore, there was no mediating effect of the type of carbohydrate load condition on the association between carbohydrate DT and *ad libitum* intake of the carbohydrate breakfast (beverage × carbohydrate DT interaction, *p* = 0.102). However, there was a weak correlation observed between pre-lunch carbohydrate DT and the amount of starch consumed in the carbohydrate breakfast beverage (*r* = 0.241, *p* = 0.041), where participants who consumed a greater quantity of starch from the breakfast beverage were more likely to have attenuated sensitivity to carbohydrate taste during the subsequent pre-lunch DT measure. No associations were observed between pre-breakfast carbohydrate DT and intake of the carbohydrate breakfast beverage. 

### 4.6. Satiety Ratings

The amount of breakfast beverage consumed, the type of breakfast beverage consumed and RE status were all assessed as potential covariates in the model to determine the relationship between carbohydrate DT and satiety ratings. None of these covariates or their interactions with DT had a significant mediating effect on the relationship between carbohydrate DT and satiety ratings (*p* > 0.05), hence, they were not included in the final model.

No significant associations were observed between carbohydrate DT and any of the ratings for fullness or hunger (pre-breakfast, post-breakfast, pre-lunch, post-lunch, or the differences between pre- and post- ratings) (*p* > 0.05).

### 4.7. Buffet Food Intake

There was a significant effect of the amount of breakfast beverage consumed on buffet intake (*p* < 0.004 for all buffet analyses), but no effect of the type of breakfast beverage (*p* > 0.05). This suggests that the amount of beverage consumed may have influenced buffet food consumption, either as a mediator of carbohydrate taste sensitivity or independently. In addition, RE status and its interaction with carbohydrate DT also had a significant effect on the buffet intake analyses (*p* < 0.036). Therefore, breakfast beverage intake (g), RE status and their interactions with carbohydrate DT were included in the final model as fixed variables.

Significant associations were observed between carbohydrate DT and total food, energy, and all macronutrient intakes from the buffet lunch (Table 5).

## 5. Discussion

The first aim of this study was to assess the associations between carbohydrate taste sensitivity, habitual and acute food intake, perceptions of satiety, and body mass. The results seem to indicate that there was an association between carbohydrate DT and multiple dimensions of eating behaviour. Participants with a lower DT (which indicates a greater sensitivity) tended to have a greater affinity for carbohydrate intake according to their self-reported 24-h diet records. Specifically, each decrease in DT concentration step was, on average, associated with an increased energy intake by 1068 kJ (i.e., an individual with a DT of 6 would, on average, consume 1068 kJ more than an individual with a DT of 7 on any given day). Similar trends were seen for total carbohydrate, starch, and sugar intakes, where each decrease in DT concentration step was, on average, associated with greater intakes by 41.8 g, 21.5 g, and 20.1 g on any given day, respectively. It should be noted that more participants were required to reach statistical significance for the starch association, but the trend is clear and is similar to what has been found in previous studies [10,11,12]. The anthropometric results supported the habitual dietary intake results, where individuals with greater sensitivity had a greater body mass. Specifically, each decrease in DT concentration step was, on average, associated with increased body weight, BMI, and hip circumference by 1.65 g, 0.38 kg/m^2^, and 0.87 cm, respectively.

The intakes from the *ad libitum* buffet lunch also supported the concept that carbohydrate taste sensitivity is associated with food intake, where individuals who had a reduced DT tended to consume greater quantities of food in the acute setting. This was not limited to intakes of carbohydrate, as we observed greater intakes of all macronutrients including fat and protein, and consequently, greater intake of total food weight and energy. This is likely driven by the types of foods that were on offer during the buffet. Foods were of mixed composition, containing mixed proportions of carbohydrate, fat, and protein. Therefore, if carbohydrate taste sensitivity was facilitating increased intake of carbohydrate, then the additional fat and protein in that food may have been consumed alongside it. This demonstrates the potential compounding harm of mixed composition foods for individuals who are highly sensitive to carbohydrate taste, as the carbohydrate in the food may be a vehicle for increased consumption of potential unhealthy food components such as saturated fat, sugar, and salt. The mixed composition of the buffet foods was a limitation of this study and any attempt to replicate this study should provide foods that are of mostly single macronutrient composition to confirm that carbohydrate taste sensitivity directly affects carbohydrate intake. Further to the buffet results, the satiety ratings did not seem to be influenced by carbohydrate taste sensitivity. This is an expected finding as it confirms that all individuals in the study were eating the buffet foods until they were comfortably full, and there was less potential that over- or under-consumption occurred. Since the individuals with higher sensitivity require greater amounts of carbohydrate before reaching a state of satiety, this suggests that these individuals have an attenuated satiety response from carbohydrate intake, as hypothesised.

Altogether, these results demonstrate that greater carbohydrate taste sensitivity is associated with greater intakes of carbohydrate and overall energy, as well as greater body mass, in line with what has been found in previous studies. In the study by Low et al., maltodextrin DT was reported to be associated with greater *ad libitum* intake of a carbohydrate-based milkshake [10] and habitual energy intakes based on 24-h diet records [12], where individuals with greater sensitivity to maltodextrin consumed higher quantities of the milkshake and had greater habitual energy intakes. A noteworthy point of difference is that the current study used oligofructose in the carbohydrate DT test rather than maltodextrin, although maltodextrin and oligofructose DT are highly correlated (*r* = 0.94) [15], indicating that they access the same peripheral mechanism. One study by Low et al. that assessed oligofructose DT did not observe any statistical relationships between oligofructose DT and energy intake or body mass [12]. Individuals who were more sensitive to oligofructose did tend to have higher energy intakes and body mass; hence, the study may have required more power to identify these associations. Interestingly, in that same study, it was found that individuals who were more sensitive to oligofructose based on ST, as opposed to DT, had greater starch intakes and waist circumference [12]. While these associations were not observed in the current study, it does seem that carbohydrate sensitivity is related to food behaviours and body mass, regardless of how the taste is perceived.

Of note, the direction of carbohydrate taste sensitivity driving carbohydrate intake was the opposite to what has been observed in fat taste studies, which is another alimentary taste with a substantial research base [30]. Individuals who are more sensitive to fat taste tend to consume reduced quantities of energy and fat in their diet, in both habitual and acute settings. [13,21,23,31,32,33,34,35,36,37]. This is because fat taste leads to a trigger of the satiety cascade, which reduces subsequent intake. To understand why these tastes differ in their regulatory mechanisms, further research about carbohydrate taste sensitivity is needed. Specifically, an understanding of the changes to carbohydrate taste sensitivity based on long-term dietary intake will help demonstrate how the regulation of carbohydrate intake is managed at a macro level. In the current study, we observed a weak association between the pre-lunch carbohydrate DT and the amount of carbohydrate consumed during the breakfast preload. This suggests that carbohydrate DT may be malleable within an individual to some extent, but further research regarding the oral mechanisms of carbohydrate taste sensitivity is necessary. Notably, no putative receptors have been identified, although it is known that carbohydrate taste receptors are independent of the receptors involved in sweet taste, TAS1R2 and TAS1R3 [15].

The second aim of this study was to assess the reliability of the carbohydrate DT test within and across days. In general, without regard for day and time, the six measures appeared to have similar agreement (ICC = 0.85), similar to what was reported by Low et al. [15] (ICC = 0.95). However, when looking at only two measures at different times, it seems that carbohydrate DT was not stable within a single day meaning that DT likely changes over the span of 3 h. Measures were not reliable until triplicate measures were taken within a given day. This suggests that at least three DT tests are required in a given day to get a reliable measurement of an individual’s daily carbohydrate DT. Furthermore, this suggests that there may be some diurnal rhythm in carbohydrate taste sensitivity. Future research could investigate how carbohydrate taste sensitivity changes throughout a day and what the health implications of that are. Testing at the same time across separate days had good agreement with only one measure, particularly for the pre-lunch measure (ICC = 0.86). We recommend that testing multiple measures across different days should aim for the same time of day. Interestingly, the two measures of carbohydrate ST intensity had poor agreement (ICC = 0.43) despite being conducted at the same time of day, which is less reliable than what was reported by Low et al. (ICC = 0.51–0.94) [15]. This seems to be in line with the agreement of ST measures for salty and umami tastes. We suggest more than two measures of carbohydrate ST for a reliable measure of an individual’s taste sensitivity.

Some limitations of this study should be noted when interpreting the results. First, a considerable number of diet records were excluded from analysis due to underreporting or being incomplete. Therefore, the habitual dietary data may not be an accurate reflection of the true dietary intake of some participants. Second, the tastant used for carbohydrate DT was oligofructose. Oligofructose is not commonly found in many foods that would be considered carbohydrate rich. Therefore, caution must be used when generalising oligofructose as a marker of carbohydrate taste that may drive the intake of carbohydrate-rich foods that do not contain oligofructose. Third, this study made some conclusions on the relationship between carbohydrate taste and body mass. However, the majority of participants recruited into this study had a healthy body weight. Therefore, these results may not be able to be generalised to an overweight or obese population, and more research on individuals with overweigh and obesity is needed.

## 6. Conclusions

Carbohydrate taste sensitivity appears to be related to eating behaviour, where individuals who are more sensitive tend to consume greater quantities of carbohydrate and energy. This increase in carbohydrate intake may further drive the intake of other components of food that have negative health outcomes, such as fat and sugar. Similarly, individuals who are more sensitive tend to have a greater body mass. The oral mechanisms that regulate carbohydrate taste sensitivity are still unclear. Further research should be conducted to identify the mechanisms that regulate carbohydrate taste sensitivity.

## Figures and Tables

**Table 1:** Suprathreshold concentrations, mean intensity ratings and intraclass correlation coefficients (ICC) for salty, sweet, sour, bitter, umami and carbohydrate tastes.

Taste	Stimulus	Concentration				Session *			
		Weak	Medium	Medium-Strong	Strong	1	5	ICC (95% CI)	*p*
Sweet	Sucrose (mM)	100	200	-	400	49.9 (22.2)	46.9 (20.0)	0.76 (0.52–0.88)	<0.001
Sour	Citric acid (mM)	1.0	3.0	-	7.0	51.5 (17.0)	55.3 (15.9)	0.78 (0.57–0.89)	<0.001
Salty	NaCl (mM)	100	200	-	400	57.7 (18.5)	57.4 (19.8)	0.45 (−0.09–0.72)	0.043
Bitter	Caffeine (mM)	1.0	2.0	-	4.0	35.7 (19.0)	41.1 (19.2)	0.66 (0.34–0.82)	0.001
Umami	MSG (mM)	3.0	6.0	-	12.0	39.9 (20.0)	45.2 (19.4)	0.49 (0.01–0.74)	0.024
Carbohydrate	Oligofructose (g/L)	36	63	112	200	30.9 (13.6)	38.7 (15.9)	0.43 (−0.05–0.70)	0.032

* Session values are combined LMS intensity ratings ± SD for each tastant. ICC indicates the agreement between session 1 and 5 LMS intensity ratings. ICC estimates, 95% CIs, and *p*-values were calculated on the basis of an absolute-agreement, two-way mixed-effects model.

**Table 2:** Aggregate results for demographic, anthropometric, carbohydrate detection threshold and Three Factor Eating Questionnaire data.

Variable	Mean (SD)	Range
Age (years)	28.5 (8.9)	18–55
Waist circumference (cm)	82.2 (11.1)	68–123
Hip circumference (cm)	97.3 (6.9)	87–118
Weight (kg)	66.6 (13.1)	49–107
BMI (kg/m^2^)	23.6 (3.4)	19–35
Pre-breakfast carbohydrate DT (step)	7.6 (2.5)	1–12
Pre-lunch carbohydrate DT (step)	7.2 (2.7)	1–12
Mean carbohydrate DT (step)	7.4 (2.1)	1–12
TFEQ—cognitive restraint	10.5 (2.4)	6–17

TFEQ—Three Factor Eating Questionnaire; cognitive restraint was scored out of 21. Individuals who scored greater than 12 on the cognitive restraint construct were categorised as restricted eaters (REs) (*n* = 7).

**Table 3:** Test-retest reliability of carbohydrate detection thresholds within day and across days.

	ICC	95% CI	*p*	Reliability
All measures	0.85	0.76–0.91	<0.001	Good
Within a day (session 2)	0.47	−0.06–0.73	0.035	Poor
Within-day duplicate measures (sessions 2 and 3)	0.60	0.20–0.80	0.005	Moderate
Within day triplicate measures	0.76	0.53–0.88	<0.001	Good
Across days pre-breakfast	0.54	0.19–0.75	0.004	Moderate
Across days pre-lunch	0.86	0.75–0.92	<0.001	Good
Across-day average	0.83	0.70–0.91	<0.001	Good

ICC estimates, 95% CIs and *p*-values were calculated on the basis of an absolute-agreement, two-way mixed-effects model. Within day = breakfast vs. lunch. All sessions were included in the analysis unless stated otherwise. Poor, moderate, and good reliability indicators were categorised as values less than 0.5, between 0.5 and 0.75, and between 0.75 and 0.9, respectively [28].

**Table 4:** Mean intakes of habitual energy and macronutrients as reported by the 24-h dietary records and their associations with carbohydrate taste detection threshold.

Variable	Mean (SD)	β	95% CI	*p*
Energy (kJ)	11,369 (4074)	−1068	−1959, −178	0.019
Total Carbohydrate (g)	296 (118)	−41.8	−68.6, −15.0	0.003
Sugar (g)	101 (58)	−20.1	−31.2, −9.2	<0.001
Starch (g)	194 (93)	−21.5	−45.2, 2.1	0.074
Protein (g)	111 (35)	1.4	−7.2, 10.0	0.742
Fat (g)	110 (68)	−10.3	−26.4, 5.8	0.206

β, 95% CIs and *p*-values were estimated using a linear mixed model including carbohydrate DT and session number as fixed variables, individual subjects set as a random variable, and RE status, including all interactions with carbohydrate DT, as covariates.

**Table 5:** Mean intakes of food, energy and macronutrients during the *ad libitum* buffet lunch and their associations with carbohydrate taste detection threshold.

	Mean (SD) Intake	β	95% CI	*p*
Food weight (g)	936.0 (287.6)	−76.1	−131.7, −20.6	0.008
Energy (kJ)	5003 (1707)	−458	−811, −105	0.011
Protein (g)	37.3 (13.7)	−3.4	−6.3, −0.5	0.021
Fat (g)	54.8 (20.3)	−5.1	−9.3, −0.9	0.018
Carbohydrate (g)	130.1 (44.2)	−12.0	−21.1, −2.9	0.028
Starch (g)	85.1 (27.7)	−7.1	−12.9, −1.4	0.015
Sugar (g)	44.6 (19.7)	−4.7	−8.7, −0.6	0.025

β, 95% Cis, and *p*-values were estimated using a linear mixed model including carbohydrate DT and session number as fixed variables, individual subjects set as a random variable, and breakfast beverage intake and RE status, including all interactions with carbohydrate DT, as covariates.

## Data Availability

Data can be obtained upon request from the CASS Food Research Centre www.cassfood.com.au.
